# Role of Nutrition in Gastroesophageal Reflux, Irritable Bowel Syndrome, Celiac Disease, and Inflammatory Bowel Disease

**DOI:** 10.1016/j.gastha.2023.06.010

**Published:** 2023-07-05

**Authors:** Amisha Ahuja, Matt Pelton, Sahil Raval, Keerthana Kesavarapu

**Affiliations:** 1 Department of Gastroenterology and Hepatology, Temple University Hospital, Philadelphia, Pennsylvania; 2 Department of Medicine, Rutgers Robert Wood Johnson Medical School, New Brunswick, New Jersey; 3 Department of Medicine, St. Peters Hospital, New Brunswick, New Jersey; 4 Department of Gastroenterology and Hepatology, Rutgers Robert Wood Johnson Medical School, New Brunswick, New Jersey

**Keywords:** Nutrition, Gluten free diet, Fodmap, Lactose free, Gastroenterology disease, Reflux, IBD, IBS, Celiac, Nonceliac gluten sensitivity

## Abstract

There remains a paucity of data on the efficacy of nutritional interventions in luminal gastrointestinal disorders. This review appraises the evidence supporting dietary modification in gastroesophageal reflux disease (GERD), irritable bowel syndrome, Celiac disease, and inflammatory bowel disease.

Alhough the use of elimination diets; high fat/low carb; low fermentable oligosaccharides, disaccharides, monosaccharides and polyols; and lactose-free diets in GERD have been studied, the evidence supporting their efficacy remains weak and mixed. Patients with GERD should avoid eating within 3 hours of lying recumbent.

Studied dietary interventions for disorders of gut-brain interaction include low fermentable oligosaccharides, disaccharides, monosaccharides and polyols and gluten-restricted and lactose-free diets. While all can be effective in carefully, individually selected patients, the evidence for each intervention remains low.

In patients with inflammatory bowel disease, enteral nutrition is established in pediatric populations as useful in reducing inflammation and partial enteral nutrition has a growing evidence base for use in adults and children. Specific carbohydrate diets and the Crohn’s disease exclusion diet show promising evidence but require further study to validate their efficacy prior to recommendation.

Overall, the evidence supporting nutritional therapy across luminal gastrointestinal disorders is mixed and often weak, with few well-designed randomized controlled trials (RCTs) demonstrating consistent efficacy of interventions. RCTs, particularly cross-over RCTs, show potential to compare dietary interventions.

## Introduction

The clinical role of nutrition in luminal disorders of the gastrointestinal (GI) system is evolving as nutritional science develops. There is increasing interest in the role of lifestyle modification in disease, but there remains a paucity of data on the efficacy of these interventions. This review aims to appraise the evidence concerning the relationship between dietary modification and gastroesophageal reflux disease (GERD), irritable bowel syndrome (IBS), Celiac disease (CeD), and inflammatory bowel disease (IBD).

## Gastroesophageal reflux

GERD presents with a myriad of clinical symptoms due to retrograde reflux of gastric contents into the esophagus. It remains the most prevalent GI disorder in the United States, affecting 18%–28% of North Americans.^1^^,^^2^ Lifestyle modifications, prescribed as either primary therapy or as adjunctive therapy to antireflux medications, include elimination of dietary triggers, alterations to meal size and timing, modification of macronutrient composition, weight loss, smoking cessation, and postural changes.^3^^,^^4^

### Elimination diets

Various foods and beverages have been implicated in the exacerbation of GERD symptoms including coffee, carbonated beverages, alcohol, acidic foods, chocolate, and spicy food. Recent reviews have outlined some of the proposed mechanisms for avoidance of dietary triggers, although the evidence is weak, and these triggers have not always correlated with GERD symptoms in prior studies^5^, ^6^, ^7^, ^8^ (Table 1).

**Table 1:** Specific Triggers and Objective Changes in GERD Monitoring, Reprinted From Newberry et al^7^

	Reduction in LES pressure	Increased gastric acid production	Decreased gastric motility	Decreased intraesophageal pH	Increased abdominothoracic pressure gradient
Coffee	X	X			
Carbonated beverages				X	X
Alcohol	X	X	X		
Acidic foods and beverages				X	
Chocolate	X				
Spicy food		X			
Dietary fat			X		
Dietary carbohydrate	X	X			
High-volume meal		X	X		
Late onset meal	X	X			

LES, Lower Esophageal Sphincter.

It is possible that concomitant avoidance of dietary triggers may assist in symptom control. A recent analysis conducted among 42,955 subjects in the Nurses’ Health Study cohort found that normal body mass index (BMI), less than 2 cups of caffeine or soda daily, and a prudent diet (higher in fruits, vegetables, white meat, and seafood) were independently associated with decreased GERD symptoms.^9^ While compelling, the study was limited to self-reported symptoms in an entirely female population without objective measures of GERD burden and may not be generalizable. Another prospective analysis of subjects from the Nurses’ Health Study found that 6 servings of coffee, tea, and soda were associated with increased reflux symptoms compared to milk, juice, and water, suggesting a dose-dependent relationship may play a role.^10^ Yet, other putative dietary triggers, including chocolate, tomato products, mint, and spicy foods (which are postulated to induce reflux through direct mucosal irritation) have not shown convincing data.

Nevertheless, clinically, patients often report relief with abstinence from certain foods. This was affirmed in a recent prospective study conducted among 100 participants with typical reflux symptoms.^11^ A majority (85%) were able to identify at least one trigger for their symptoms and, using a standardized survey, heartburn and regurgitation symptoms improved at 2 weeks after cessation of culprit. While the placebo effect may confound these results, such findings provide compelling evidence for the avoidance of foods on an individualized basis. While the evidence for blanket avoidance of implicated trigger foods is limited and mixed, elimination diets may have the largest role when individualized and closely followed in the outpatient setting to ensure safety^12^ (Table 2).

**Table 2:** Level of Evidence and Efficacy of Diets Tested in GERD

	Level of evidence	Results	Limitations	Citations
Elimination diets^a^	Observational, RCT	Mixed	Limited objective measures, high variability in triggering foods	Mehta^9^^,^^10^, Tosseti^11^
High fat/low carb	Observational, RCT	Positive	No objective change in pH for high vs low fat	Pointer,^13^ Gu,^14^ Fox^15^
Low FODMAP	RCT	Mixed	No objective change in pH	Riviere^16^
Mediterranean	Observational	Positive	No objective measures	Mone^17^
High fiber	Observational	Positive	Decrease in total reflux events and maximal reflux time but not esophageal pH	Morozov^18^
Lactose-free	Subgroup analysis of RCT,	Negative	No objective measures	Fernando^19^

a Elimination diets are inherently heterogenous and refer to reduced foods patients associate with symptoms—some of these interventions overlap with other restricted diets present in the table.

### Macronutrient composition

Recently, attention has been paid to the effects of macronutrient composition on GERD symptoms. High fat diets, including greasy foods, for example, have long been anecdotally implicated with reflux symptoms. Yet, data remain mixed with contradictory associations. There is growing interest in the role of carbohydrates in GERD and data to support lesser intake as a part of therapy. It is postulated that absorption of carbohydrates leads to a fermentation process that, by way of a neurohormonal release, contributes to lower esophageal sphincter relaxation.^12^

Several observational and randomized trials investigating carbohydrates provide further data on the topic. For example, significant reduction of reflux symptoms was seen after institution of a high-fat, low-carbohydrate diet in a study of 144 obese women with a diagnosis of GERD.^13^ More recently, a single-blind, randomized controlled trial (RCT) was performed among 95 participants with GERD with 1 of 4 combinations of total carbohydrate (low or high) and simple carbohydrates (low or high) dietary interventions for a 9-week period using a high carbohydrate/high simple carbohydrate diet as a control.^14^ All 3 treatment groups with some limitations of carbohydrates were associated with fewer reflux symptoms and fewer documented episodes of elevated pH, with high total/low simple carbohydrate diet showing the most significant reduction. Given that results could be achieved in this short treatment interval seems to push back against arguments that carbohydrate reduction results in GERD improvement through weight loss alone (although this may certainly be contributing) and offers a potential therapeutic avenue for patients suffering from cumbersome symptoms.

This suggests that the type of carbohydrate plays a role in symptom modulation. A high fiber or complex carbohydrate diet may help with GERD through improvement of lower esophageal sphincter resting pressure. In a study of 36 patients with nonerosive reflux disease, those consuming high-fiber diets had reduced reflux symptoms without an effect on objective measures of acid exposure.^18^ Additional studies are warranted to better understand these findings and tease out the discrepancy between symptomology and objective pH measures.

### Meal timing

Meal timing around periods of sleep has been implicated in reflux and the American College of Gastroneterology recommends minimum of 3 hours of lag time prior to lying flat.^3^ This is supported by a prospective, unblinded crossover RCT enrolling 32 patients with typical reflux symptoms compared patients consuming a standard meal either at 6 hours or 2 hours prior to going to bed. Late evening meal consumption demonstrated significantly more supine reflux on pH monitoring when compared to early meal consumption.^20^ This reliable, undisputed dietary advice is one of the most accessible interventions for all patients and should be routinely recommended shortly after diagnosis.

### Low FODMAP

A recent-open labeled RCT among 31 patients with proton pump inhibitor–refractory GERD assessed the efficacy of a low fermentable oligosaccharides, disaccharides, monosaccharides and polyols (FODMAP) diet compared to standardized dietary and lifestyle advice. At 4 weeks, although symptoms improved subjectively in both groups, no difference in response between groups was seen without any improvement in postintervention pH testing in both groups.^16^ Limited benefit from FODMAP diet in proton pump inhibitor–refractory GERD findings coupled with the restrictive nature of this dietary pattern do not make this a viable recommendation for patients currently unless further testing reveals greater benefits over less restrictive dietary strategies.

### Summary of dietary guidance for GERD patients

Although RCTs have been conducted on the use of elimination diets, high fat/low carb, low FODMAP, and lactose-free diets in GERD, the evidence supporting their efficacy remains weak and mixed. The evidence supporting their efficacy is largely based on patient-reported symptoms without robust corresponding objective measures of reflux.

## Functional bowel disorders

Disorders of gut-brain interaction (DGBI), simultaneously heterogenous and overlapping, are symptomatic diagnoses of exclusion organized under Rome IV criteria by shared pathophysiologic changes: motility disturbance, visceral hypersensitivity, altered mucosa or immune function, altered gut microbiota, and altered central nervous system processing.^21^ Diet plays an important role in these disorders: Food and its byproducts directly interface with the luminal surface of the gut, influencing gut distension and contributing to local inflammation.^22^ In a recent survey-based study in IBS patients, 84% of patients reported at least one food trigger to their symptoms, and patients with more severe symptoms reported several food triggers.^23^ Yet, there are also some contradictory data suggesting that after periods of abstinence from a food perceived as a trigger, rechallenging patients with that food unreliably reproduces symptoms.^24^ Nevertheless, patients with functional bowel disorders are particularly eager for dietary interventions, with one recent study demonstrating that it is the preferred therapeutic modality for a cohort of IBS patients.^25^

Despite the lay interest in diet as therapy and the putative role it plays in bowel symptoms, data regarding its efficacy in DGBI remain relatively sparse. Some of this is due to the difficulty of conducting trials related to diet with placebo and nocebo effects potentially playing a large role in therapeutic outcomes.^26^ Additionally, disease categories under the DGBI umbrella are fluid, with overlapping symptoms existing across multiple disorders. Although dietary recommendations are often made on the basis of experience and physiologic understandings of disease, clear outcome data may not always be readily apparent or appreciable due to the fact that these disorders may exist on a spectrum.^27^ There are a few notable exceptions to the paucity of dietary treatment data for DGBI; these exceptions include low FODMAP, lactose-free, gluten-free, and high fiber diets, which are associated with decreased symptoms in observational studies (Table 3).

**Table 3:** Level of Evidence and Efficacy of Diets Tested in DGBI

	Level of evidence	Results	Limitations	Citations
Low FODMAP	RCTs, systematic review	Positive	Limited long-term follow-up	Böhn,^28^ Eswaran,^29^ Halmos,^30^ Wang^31^
Gluten-free	RCTs, systematic review	Mixed	Poor study retention, low n, large nocebo response	Shahbazkhani,^32^ Yu^33^
Lactose-free	Case-control, RCT	Negative	Self-reported symptoms, confounded by FODMAPs	Varjú,^34^ Moritz^35^
High fiber	RCTs, systematic reviews	Positive	Limited long-term follow-up and IBS subgroup analysis	Moayyedi^36^

### Low FODMAP diet

FODMAPs or short-chain carbohydrates are poorly absorbed and osmotically active in the intestinal lumen. Their influence on bowel symptoms is thought to be secondary to distension due to short-chain fatty acids, gas release, and osmotic effects. They also may contribute to dysbiosis and local mast cell activation resulting in epithelial barrier dysfunction and hypersensitivity.^37^

Society guidelines recommend that implementation of a low FODMAP diet occurs in 3 phases (1) eliminating foods high in FODMAPs, (2) gradual reintroduction of these foods while monitoring symptoms, and (3) personalization of the diet to avoid the patient’s specific dietary triggers.^38^ The latter 2 phases are important given how restrictive a low FODMAP diet can be, highlighting the importance of appropriate patient selection and a low threshold of diet cessation should there be no symptom improvement within 6 weeks. There are also little long-term data on how such changes can alter microbiota concentrations. This is concerning, as alterations in the microbiome are hypothesized to contribute causatively to the development of IBS symptoms. A recent systemic review demonstrated that a low FODMAP diet decreased concentrations of *Bifidobacteria*, which has been linked to IBS-symptom severity. However, microbiome constitution was not holistically affected overall in these 9 prospective trials, the longest of which lasted 3 months.^39^

While there still is a paucity of long-term outcome data on FODMAP restriction and its impact of the microbiome, the diet is efficacious for symptom and disease-specific quality of life improvement, particularly in those patients with IBS-D.^28^, ^29^, ^30^, ^31^ Additionally, the low FODMAP diet may have synergistic effects with probiotics, particularly those with *Lactobaccillus*.^40^ Given the evidence base for this dietary intervention, a limited trial supplemented by guidance from a dietician is the first line intervention in appropriate IBS patient populations.

### Gluten-free diet

Guidelines recommend that all patients with IBS, a multifactorial disorder, be ruled out for concomitant CeD, because presenting symptoms for each of these disorders are often overlapping.^41^ Even after ruling out true CeD, nonceliac gluten sensitivity (NCGS), defined as the improvement in intestinal and extra-intestinal symptoms with gluten avoidance, can be hard to parse out from symptoms of IBS.^42^ Nonetheless, patients with isolated IBS report improvement in symptoms via gluten avoidance and a few RCTs have reported a benefit of gluten avoidance in IBS patient populations. Mechanistically, there is some thought that gluten itself may trigger IBS symptoms through decreased expression of tight junction proteins and increased intestinal permeability.^43^

However, an inherent difficulty to interpreting the impact of a gluten-free diet (GFD) results from its low FODMAP nature; gluten and FODMAPs are concurrently present in foods such as wheat, barley, and rye. Thus, in reducing gluten, one is also reducing FODMAPs. Ultimately, more information is needed to clarify the role of gluten-restriction/avoidance on the improvement in lower GI symptoms and little evidence supports recommending an empiric trial of gluten avoidance in IBS patients.

### Lactose-free diet

Similarly, lactose is itself a member of the FODMAP class and may trigger IBS symptoms in patients. Symptoms of lactose intolerance also overlap with those of IBS, which makes the relationship between the 2 particularly difficult to parse out. Lactose-free or restricted diets have been studied in IBS patients, as they self-report lactose intolerance more than healthy controls. However, a recent meta-analysis of case-control studies found that when measured with objective testing (lactose-breath test, lactose tolerance test, or genetic testing), IBS patients did not have significantly more lactose maldigestion when compared to healthy controls.^34^ Those IBS maldigestors, however, may experience symptoms at lower doses of lactose than healthy controls. It is reasonable for all patients with lower GI disorders to trial lactose avoidance, although society guidelines do not offer this in any specific algorithms.

### Fiber-rich diet

Fiber can impact bowel symptoms through several mechanisms, including increasing stool bulk, viscosity, transit time, and fermentation rates.^44^ Such wide effects also make for wide applicability, offering potential symptom amelioration for patients with functional diarrhea, constipation, and IBS subtypes. A meta-analysis of 14 RCTs found that soluble fiber treatment leads to global symptomatic improvement in patients with IBS with little harm.^36^ These findings have led to society recommendations for the use of soluble fibers in this patient population.^41^

Specific foods often help with symptoms partly due to their fiber content, including aloe, kiwifruit, figs, and prunes.^37^ A few RCTs have been conducted, although they have generally been small without objective measures of gut function: 3 studies on aloe vera and a single study on prunes.^45^^,^^46^ Most convincingly, a comparative effectiveness RCT showed similar efficacy in patient-reported outcomes between psyllium fiber, kiwifruit, and prunes when compared to patient’s baseline bowel habits.^47^ Although RCTs investigating these specific high-fiber foods exist, they are few and far between, with an overall lack of data on the efficacy and dose-dependent responses. Thus, if patients cannot tolerate fiber supplementation in the form of psyllium or favor traditionally natural remedies, supplementation through specific high fiber, low FODMAP foods may be a reasonable approach.

### Summary of dietary guidance for DGBI patients

Studied dietary interventions for DGBI include low FODMAP, gluten-restricted, and lactose-free diets. Each can be effective in carefully, individually selected patients. Of the 3, the FODMAP diet is the most thoroughly evaluated; however, the strength of the evidence for each intervention remains low. When trialing any of these diets, patients should be closely monitored by a trained professional dietician. Outside of elimination diets, soluble fiber supplementation can improve bowel function and global symptoms.

## Celiac disease and nonceliac gluten sensitivity

### Celiac disease

CeD is an immune-mediated enteropathy triggered by ingestion of gluten in susceptible individuals. With genetic predisposition and exposure to gliadin, a soluble protein component in gluten found in wheat, rye, and barley, patients develop an adaptive and innate immune response. Once diagnosis is established, the standard of care treatment is an initiation of a GFD. While generally well tolerated, following a GFD is difficult and requires regimented monitoring by self and family to ensure all foods, including those from restaurants or grocery stores are gluten-free.

### Gluten-free diet

Avoidance of the principal sources of dietary gluten present in wheat, rye, and barley is pivotal to a GFD (Table 4). An ideal GFD should consist of naturally gluten-free (GF) foods (eg, unprocessed meat, dairy, vegetables, nuts, and fruits and grains) and/or commercially manufactured GF products consisting of < 20 ppm of gluten. However, adhering to this diet poses nutritional implications. To make GF products more palatable, consistent, and retain the physical and sensorial properties of gluten-containing products, additional carbohydrates, lipids, and calories are often added along with gluten replacers such as xanthan and guar gum, hydrocolloids, and enzymes. This processing results in GF products having a poor nutritional profile contributing to micronutrition deficiencies and abnormal macronutrient composition promoting metabolic disease.^49^

**Table 4:** Grains, Seeds, and Other Starches Sources in GFD

Naturally gluten-free grains	Naturally gluten containing grains
Amaranth	Wheat (spelt, semolina, and durum)
Arrowroot	Rye
Buckwheat	Barley
Corn/Maize	Triticale
Indian rice grass	Kamut
Legumes mesquite	
Millet	
Nutes	
Potato	
Quinoa	
Rice	
Sorghum/Milo	
Soy	
Tapioca	
Tef/Teff	
Wild rice	

With the combination of poor nutritional profile of manufactured GFD and improvement of GI-related symptoms with initiation of GFD, concern has been raised about patients with CeD becoming overweight or obese.^50^ Proposed mechanisms of weight gain include macronutrient composition of GF food and recovery of mucosal functionality. However, evidence on how significant this weight gain is mixed. For example, a large cohort study on 679 subjects following a GFD for 39 months found that 15.8% of subjects moved from normal/low BMI to an overweight BMI and 22% that were overweight at diagnosis gained weight with a 2-point or more rise in BMI.^51^ On the other hand, a recent systematic review and meta-analysis of weight gain associated with GFD found that patients were not at higher risk of becoming overweight or obese.^52^ As the explosion of available GF products coincides with rising international obesity, close monitoring of weight in patients on GFDs should continue.

While GFDs may have higher caloric density and help recover luminal regularity, processed GF products are generally not enriched or fortified. A review of available GF cereal products showed 81% contained lower amounts of folate content, 14% had adequate levels, and only 5% of cereals were fortified with folic acid. Furthermore, 77% had lower iron content and none of the bread or pasta products were enriched with iron.^53^ Similarly, GF cereals tend to have 30% lower dietary fiber content with 30% compared with their gluten-containing counterparts.^53^

To overcome the dietary composition of GF products, providers should encourage patients to consume grains, seeds, legumes, and nuts that are naturally GF. Inclusion of these grain products provided a higher nutrient profile compared to the standard GF dietary pattern with higher levels of protein, iron, calcium, and fiber.^54^ If consuming GF products, choose enriched/fortified GF flours, breads, pastas, and cereals along with consumption of noncereal sources of folate, iron, and fiber. Patients following a strict GFD may benefit from the addition of a vitamin and mineral supplement, particularly if the patient is deficient.

Current evidence-based guidelines suggest that patients should be referred to a dietician with experience in CeD to assist in dietary plan design, educate on reading food labels, and ensure patients follow a nutritionally adequate GFD. Gonzalez et al showed that patients following with a dietician increased plant-based foods as well as decreased sugar content, red meat, and ultra-processed food intake (*P* < .001), which correlated with short-term improvements in body composition in pediatric celiac patients.^55^^,^^56^ Of note, patients following with a dietician consumed higher fiber diets, resulting in 80% reduction of constipation (*P* < .001).^56^

### Lactose-free diet

Injury to small intestinal vili can cause malabsorption of lactose. Therefore, a limited lactose intake in CeD during the initial weeks to months of diagnosis and dietary therapy is recommended. Once restoration of mucosa occurs, most patients can often tolerate lactose. However, lactose intolerance has been established as a cause of persistent GI symptoms in 8% of patients with CeD are nonresponders to a GFD.^57^ These patients may need to remain on a lactose-restricted diet in addition to GFD (Table 5).

**Table 5:** Level of Evidence and Efficacy of Diets Tested in CeD

	Level of evidence	Results	Limitations	Citations
Celiac disease				
Gluten-free	Unequivocal	-	-	-
Lactose-free diet + GFD	Observational	Positive	Limited evidence (extremely low n), very restrictive, only PRO	Dewar^57^
Low FODMAP + GFD	RCT	Positive	Trialed for 21 d, no long-term follow-up, very restrictive, only PRO	Roncoroni^58^ van Megen^59^
Nonceliac gluten sensitivity				
Gluten-free	Cross-over RCT	Mixed	After 2 y GFD, patients unable to reliably distinguish GFD from non-GFD	Zanini^60^
Low FODMAP + GFD	Observational, cross-over RCT	Mixed	Evidence limited, significant recall bias and self-selection	Dieterich^61^ Biesiekierski^62^

### Low-FODMAP diet

Foods containing FODMAPs can trigger IBS-like symptoms in patients with or without CeD. One double-blind RCT suggests that low-FODMAP diet in patients with CeD on GFD with persistent symptoms may improve abdominal pain and stool consistency when compared with those on unrestricted FODMAP GFD. Short-term low FODMAP GFD can help improve quality of life and GI symptomology in patients with CeD.^58^^,^^59^ While compelling initial results, further RCTs evaluating the long-term viability and impact of this combination are lacking and would be essential to validate its use. Of note, additional restrictions like low-FODMAP diet can exacerbate underlying nutritional deficiencies; therefore, careful nutritional surveillance and counseling is mandatory.

### Nonceliac gluten sensitivity

NCGS is used to describe individuals who have intestinal and/or extraintestinal signs and symptoms induced by gluten ingestion that improve when gluten-containing grains are removed from the diet and when CeD and wheat allergy have been ruled out.^63^ The true prevalence is unknown due to the lack of definitive diagnosis criteria and overlap with IBS. While the exact causal agent of NCGS is unknown, there are several components of wheat that are potentially implicated including gluten, FODMAPs, alpha amylase trypsin inhibitors (AATI), and wheat germ agglutinins.

Given the overlap in symptoms between CeD and gluten sensitivity, CeD must be excluded with serologic and histologic evidence prior to diagnosis. The third International Expert Meeting on Gluten-Related Disorders recommends confirming a diagnosis of NCGS using the Salerno criteria. Those not meeting this criterion should be investigated for alternate etiologies of symptomology.

### Gluten-free diet

For many with NCGS, gluten avoidance is reported to improve symptomology. Given the nutritional, social, and economic implications of a GFD, it must be administered with guidance and support of a registered dietician. As such the threshold for gluten tolerance needs to be further characterized in NCGS. The literature currently includes patients with a variety of levels of intolerances; some patients require strict adherence to eliminate symptoms, while others can tolerate some levels of gluten.^64^ Therefore, it can be recommended that patients follow more of a gluten-reduced diet if tolerated to avoid the consequences of a long-term GFD.

It has been suggested to rechallenge with gluten after 1 year of following strict GFD. Interestingly, in a small RCT, gluten rechallenge in patients with NCGS adhering to a GFD-induced symptom recurrence in only a third of patients, compared with a placebo reintroduction that induced symptoms in nearly half of patients.^60^ While compelling, the size of the trial and the form of placebo warrant replication prior to drawing strong conclusions on reintroduction of gluten into these patients’ diets. Any consideration to liberalize the diet should be attempted incrementally in lockstep with a dietician. An alternative explanation for the above-trials results may come from the form of gluten reintroduced—isolated flour extract may lack other ingredients that contribute to patients’ intolerance. Thus, in patients with NCGS on long-term GFD still complaining of persistent clinical symptoms other dietary components of wheat, rye, and barley should be considered as offending agents, such as FODMAPs and AATIs.

### Low-FODMAP diet

Despite adherence to a GFD, some patients report persistent GI and extra-intestinal symptoms which may suggest alternate components of diet such as FODMAPs could also act as triggers for clinical manifestations of NCGS. For example, a double-blinded placebo-controlled crossover study in patients with self-reported NCGS on a GFD randomized to muesli bars containing placebo, gluten, or fructans found overall symptoms and, more specifically, symptoms of bloating were worse for those exposed to fructans rather than gluten. Interestingly, there were no significant differences between patient-reported symptoms between gluten and placebo exposures.^65^ This study was notably small with a sample size of 59 and lacked long-term follow up. However, this suggests that improvement in GI symptoms with gluten-avoidance for DGBI patients may be confounded in part by the inherent reduction in FODMAPs within a GFD. Similarly, FODMAP restriction improved GI symptoms including abdominal pain, reflux, and stool consistency in patients with NCGS.^61^^,^^62^ The authors concluded that fructans in wheat were likely to induce GI symptoms rather than gluten. Further work should emphasize long-term follow-up of patients’ trialing combinations of restricted FODMAPs and gluten to tease out the differences in symptom onset and severity.

### AATI

AATIs, which are plant-based proteins in wheat, serve as self-defense against pests.^66^ When consumed, these proteins are resistant to proteolytic degradation in the intestines and act as potent activators of the innate and adaptive immune response, which results in both intestinal and extraintestinal symptoms. The role of AATIs in NCGS (and IBS) is unclear with no direct evidence of the impact on the gut in vivo studies. Thus, there are no recommendations supporting empiric avoidance and further work is required to delineate relative concentrations and methods of reduction of AATIs before observational studies can be conducted in humans.^67^

### Summary of dietary guidance for CeD and NGCS patients

All patients diagnosed with CeD should see a dietician and start a monitored GFD. If symptoms persist after initiation of a GFD, the most common culprit is difficulty adhering to a GFD. If symptoms persist and adherence is confirmed, it is reasonable to trial lactose or FODMAPs restriction in addition to a GFD, although the evidence of their efficacy is limited. For patients with self-reported NCGS, a GFD can be trialed and may alleviate symptoms. If no relief is achieved, trialing the elimination of FODMAPs is reasonable, but symptom improvement may be confounding if concomitant IBS is present.

## Nutritional management of inflammatory bowel disease

IBD is a chronic, relapsing autoimmune disease characterized by inflammation of the small and/or large bowel that usually presents as abdominal pain and frequent, bloody bowel movements. It affects approximately 0.3% of citizens of North America and Europe, and its incidence is rising in nonwestern countries.^68^ While pharmacotherapy remains the mainstay of IBD management, there is a growing interest in the role of dietary interventions as a modifiable factor in disease management. Despite the diversity of dietary interventions and investigations into their efficacy, the strength of the data remains low.^69^^,^^70^ Here, we review the existing data regarding the role of dietary interventions in induction and maintenance of remission in IBD.

### Enteral nutrition

Enteral nutrition (EN) is the most widely studied nutritional intervention in IBD. While RCTs have shown EN improves flare symptoms, improves malnutrition, and reduces maladaptive intestinal immune responses in pediatric populations with Crohn's disease (CrD), it has lower rates of clinical response in adults with CrD.^71^, ^72^, ^73^ In UC, the use of EN is not well established. One of the clinical trials comparing steroids with EN showed no statistically significant difference in the rates of remission, but a higher rate of postoperative infection in the EN arm.^74^ However, new RCTs show that the use of exclusive EN along with corticosteroids may augment corticosteroid responsiveness in acute severe UC^75^ (Table 6).

**Table 6:** Level of Evidence and Efficacy of Diets Tested in IBD

	Level of evidence	Results	Limitations	Citations
EN	RCTs	Mixed	Efficacy limited to pediatric patients, poor tolerance, and compliance	Borrelli,^73^ Grover^72^
CDED plus PEN	RCTs	Positive	Requires enteral access and strict dietary guidance, limiting feasibility and compliance	Levine,^76^ Nguyen^77^
Elimination diets^a^	RCT	Mixed	Reanalysis of RCT, high degree of confounding, and no change in inflammatory biomarkers	Allenberg,^78^ Bentz,^79^ Gunasekeera^80^
Specific carbohydrate diet	RCT	Positive	Difficult to maintain diet and no change in inflammatory markers	Lewis^81^
Mediterranean	RCTs, observational	Mixed	Limited efficacy, observational design concerning for confounding, and no change in inflammatory markers	Chicco,^82^ Haskey,^83^ Lewis^81^
Low FODMAP	Systematic review, RCTs	Mixed	No change in inflammatory biomarkers, confounded by concomitant IBS symptoms, primarily PRO	Bodinin,^84^ Cox,^85^ Pederson,^86^ Zhan^87^
High fiber	RCTs	Positive	No change in inflammatory biomarkers, PRO only, small n	Brotherton,^88^ Fitsch^89^

a Heterogeneous in nature, each study eliminated: red meat - Allenberg et al,^78^ food selected based on IgG antigenetic profile.^79^^,^^80^

### Crohn's disease exclusion diet plus partial enteral nutrition

Partial Enteral Nutrition (PEN) is gaining popularity as a dietary intervention for supplementing medical IBD interventions, in which between 25% - 75% of patient caloric intake comes in the form of calorically dense liquid nutrition. For example, a meta-analysis of 4 RCTs found that after induction, higher percentages of patients maintained remission with infliximab therapy combined with PEN compared to infliximab alone.^77^ Furthermore, PEN has been shown to have similar efficacy to 6-mercaptopurine in maintaining remission in patients with CrD.^90^ There is growing interest in the combination of exclusion diets and PEN to supplement CrD treatment.

The CrD disease exclusion diet (CDED) plus partial enteric nutrition (PEN) is a whole food diet which avoids products known to have a proinflammatory effect on the intestinal mucosa, coupled with calorie-rich formula. This diet has shown promising ability to achieve corticosteroid-free remission with sustained reductions in inflammation and better tolerability than exclusive EN in a recent RCT by Levine et al.^76^ Notably, trials comparing EN with PEN with an unrestricted diet found EN to be superior at inducing remission, increasing quality of life, increasing albumin and platelets, and decreasing erythrocyte sedimentation rate.^91^ Thus, Levine proposes that PEN works only when paired with an anti-inflammatory diet, suggesting that the type of paired diet is the main driver of patient response. Further multicenter RCTs are needed to verify the efficacy of the CDED and other anti-inflammatory diets plus PEN in induction and maintenance of remission.

### Low-FODMAP diet

Low-FODMAP diets are well established in the treatment of IBS, and the diet shows promise in the treatment of IBD. Most significantly, a RCT found that a low FODMAP diet decreased calprotectin, correlated with intraluminal disease activity, and improved quality of life when compared to a standard diet in patients with inactive and active IBD.^84^ Another RCT limited to patients with quiescent IBD found the low FODMAP diet mildly reduced persistent gut symptoms but did not alter microbiome diversity or inflammatory markers.^85^ These and other investigations into the role of low FODMAP diets are confounded by the co-existence of IBS-like symptoms in IBD patients, making it difficult to parse out the true influence of the diet on IBD alone.^86^ Further studies should focus on mitigating this confounder.

### Specific carbohydrate and mediterranean diets

Specific carbohydrate diet (SCD) is a modified diet primarily excluding complex carbohydrates composed predominantly of monosaccharides, solid proteins, and fats.^92^ The underlying concept behind SCD is that undigested di-saccharides and poly-saccharides can stimulate bacterial overgrowth and can perpetuate inflammation. Initial observational studies have shown promise for the use of the SCD for decreasing C-reactive protein and improving quality of life in pediatric patients with CrD and UC. Additionally, a small RCT found that the use of the SCD shows improvement in clinical and inflammatory burden in 18 pediatric CrD patients.^93^ The diet, however, is relatively restrictive, eliminating a large amount of calorie-dense sources of carbohydrates. Interestingly, a large RCT found that in patients on stable medical therapy for IBD still experiencing mild-moderate disease activity, the SCD was comparable to Mediterranean diet in achieving symptomatic remission.^81^ While, neither diet significantly lowered patients’ C-reactive protein levels, fecal calprotectin was significantly lowered in the SCD arm.

Since the Mediterranean diet performed similarly to the SCD regarding symptomatic remission, some providers prefer the Mediterranean diet for its ease, accessibility, and established cardiometabolic health benefits.^83^ It is composed of increased consumption of fish, dairy, fruits, nuts, and vegetables with decreased intake of saturated fats, processed foods, and meat. A large observational study found patients with IBD placed on Mediterranean diets experienced improved disease activity, inflammatory markers, liver steatosis, and cardiometabolic health.^82^ Another observational study found an association between the Mediterranean diet and decreased mortality in a large cohort of IBD patients.^94^ It is important to note, however, that the head-to-head trial between the Mediterranean diet and the SCD is the highest level of evidence available, which found no improvement in fecal calprotectin and C-reactive protein in the Mediterranean diet and improvement in only the fecal calprotectin in the SCD.^81^ More high-quality evidence is required to clarify the benefits of the SCD and Mediterranean diets in the treatment of IBD.

### Fiber

An interesting difference between the Mediterranean diet and the SCD is their fiber content. While Mediterranean diets are generally high in fiber, SCDs are usually low in fiber. The role of fiber in IBD dietary interventions is controversial. Both high and low fiber diets have been trialed in observational studies, with mixed and contradictory results. Currently, weak evidence suggests that higher fiber content may be beneficial to patients with IBD. For example, Brotherton et al found that a high-fiber diet improved health-related quality of life and GI function in 7 IBD patients but did not affect inflammatory markers in a small RCT.^88^ Another cross-over observational study of 17 UC patients in remission found a high-fiber diet increased quality of life, decreased inflammatory markers, and reduced intestinal dysbiosis.^89^ As both studies were small in size with limited generalizability, further work is needed to clarify the role of fiber in IBD dietary recommendations.

### Food additives and emulsifiers

There is emerging evidence that the additive carrageenan may be related to the pathophysiology of IBD because of epidemiologic studies and animal models. As the SCD and Mediterranean diets are naturally low in carrageenan, it is hypothesized that the lack of the additive may contribute to their efficacy.^95^ One RCT investigating a low-carrageenan diet showed promise for efficacy; however, the trial was criticized for its study design, data interpretation, and data analysis.^96^^,^^97^ Furthermore, high-quality RCTs are required to validate these findings.

Various emulsifiers have been shown in animal models to induce intestinal inflammation by decreasing the diversity of the gut microbiota and increasing the proinflammatory potential. Thus, the International Organization for the Study of Inflammatory Bowel Disease released dietary guidance suggesting patients may benefit from limiting intake of emulsifiers such as carboxymethylcellulose and polysorbate-80 based on very low-quality evidence from animal models and epidemiologic studies.^98^ Further observational and interventional trials in humans are necessary to determine the role of dietary emulsifiers in IBD and the role of their dietary exclusion in the induction and remission of IBD.^99^

### Summary of dietary guidance for IBD patients

Concrete evidence-based guidelines for patients with IBD are limited by low quality of evidence.^100^ Individual tailoring of diet based on specific age group, disease type, severity, and patient preference are generally recommended for dietary guidance. In general, diets high in animal fat and processed foods or low in fruits and vegetables are associated with increased incidence and exacerbation of IBD. EN is established in pediatric populations as useful in reducing inflammation and PEN has a growing evidence base for use in adults and children. SCD and anti-inflammatory diets have also shown promising evidence, but there is a lack of generalizable evidence to incorporate into guidelines. Further high-quality research is necessary to guide the role of dietary interventions in IBD.

## Role of a dietician

The presence of dieticians, who are often part of an interdisciplinary gastroenterology practice, contributes significantly to patient satisfaction with care.^101^ Furthermore, patient adherence to diet is often higher in settings in which a dietician is available.^38^ Given the potential for avoidance diets to result in malnutrition, national guidelines recommend instituting dietary changes in conjunction with a dietician or recognizing that they may not always be available for all patients, in-depth educational resources for patients.^38^^,^^41^ Therefore, routine use of dieticians should be encouraged for GERD, IBS, CeD, IBD, and when implementing interventional dietary modification on any disorders of the GI system.

RD utilization has shown improvements in patient-related health outcomes in IBS and Celiac disease; however, limited to no outcome studies exist in patients with GERD and IBD. Participants with IBS noted reduction in symptom severity based on IBS-SSS scoring systems and QOL.^102^ Nutrition interventions implemented with an RD show improvement in adherence to GFD, food quality (ie naturally GF foods and/or enriched foods), and subsequent improvements in body composition, fat free mass, and nutritional markers.^55^^,^^103^ Further trials are necessary to determine the impact of RDs on clinical outcomes particularly in reflux and IBD.

## Conclusion

There is some evidence of the efficacy of specific diets in GERD, IBS, CeD, and IBD. However, this evidence is mixed and often weak, with few well-designed RCTs demonstrating consistent efficacy of interventions. RCTs, particularly cross-over RCTs, show potential to robustly compare dietary interventions within and across these diseases.
